# The role of the transcription factor Rbpj in the development of dorsal root ganglia

**DOI:** 10.1186/1749-8104-6-14

**Published:** 2011-04-21

**Authors:** Ze-Lan Hu, Ming Shi, Ying Huang, Min-Hua Zheng, Zhe Pei, Jia-Yin Chen, Hua Han, Yu-Qiang Ding

**Affiliations:** 1 Department of Anatomy and Neurobiology, Tongji University School of Medicine, 1239 Siping Road, Shanghai 200092, China; 2 Department of Neurology, Xijing Hospital, Fourth Military Medical University, Xi'an 710032, China; 3 Department of Medical Genetics and Developmental Biology, Fourth Military Medical University, Xi'an 710032, China

## Abstract

**Background:**

The dorsal root ganglion (DRG) is composed of well-characterized populations of sensory neurons and glia derived from a common pool of neural crest stem cells (NCCs), and is a good system to study the mechanisms of neurogenesis and gliogenesis. Notch signaling is known to play important roles in DRG development, but the full scope of Notch functions in mammalian DRG development remains poorly understood.

**Results:**

In the present study, we used *Wnt1-Cre *to conditionally inactivate the transcription factor Rbpj, a critical integrator of activation signals from all Notch receptors, in NCCs and their derived cells. Deletion of *Rbpj *caused the up-regulation of *NeuroD1 *and precocious neurogenesis in DRG early development but led to an eventual deficit of sensory neurons at later stages, due to reduced cell proliferation and abnormal cell death. In addition, gliogenesis was delayed initially, but a near-complete loss of glia was observed finally in *Rbpj*-deficient DRG. Furthermore, we found P75 and Sox10, which are normally expressed exclusively in neuronal and glial progenitors of the DRG after the NCCs have completed their migration, were co-expressed in many cells of the DRG of *Rbpj *conditional knock-out mice.

**Conclusions:**

Our data indicate that Rbpj-mediated canonical Notch signaling inhibits DRG neuronal differentiation, possibly by regulating *NeuroD1 *expression, and is required for DRG gliogenesis *in vivo*.

## Background

The nervous system is made up of a wide variety of neuronal and glial cell types. How these different cell types are generated from multipotent progenitors during development is a fundamental and largely unanswered question in neuroscience. The dorsal root ganglion (DRG), which consists of several well-characterized types of sensory neurons and glial cells, is an attractive model system to investigate the molecular processes underlying cellular differentiation in the nervous system [1]. Sensory neurons and glial cells in the DRG derive from the neural crest, a tissue that exists transiently during mammalian embryogenesis at the border between the ectoderm and the neural plate [2]. Between embryonic day (E)8.5 and E10.0 in the mouse, neural crest stem cells (NCCs) begin to exit the neural tube, and those that migrate along the ventral pathway between the neural tube and the dermamyotome form the DRG [3]. Between E9.25 and E13.5 in the mouse, NCCs first give rise to large neurons that express neurotrophic tyrosine receptor kinase (Trk)C, and then to medium-sized *TrkB*^+ ^and small-sized *TrkA*^+ ^sensory neurons [4,5]. NCC-derived glial cells are also generated during this period, including satellite cells and Schwann cells, although these begin to differentiate about 1.5 days after sensory neurons [4,6].

Many genes are involved in the generation of sensory neurons and glia from multipotent NCCs. Among the various cell-surface proteins known to be expressed by NCCs, the low affinity neurotrophin receptor P75 has been widely used to identify and isolate NCCs [7]. In addition, P75 interacts with TrkC, the high-affinity receptor for Neurotrophin-3, to promote neuronal differentiation of NCCs *in vitro *[8]. The high-mobility group transcription factor SRY (sex determining region Y) box 10 (Sox10) is expressed in pre-migratory and migratory NCCs and plays a role in maintaining the multipotency of NCCs [9]. Expression of *Sox10 *turns off in daughter cells committed to neuronal fates, but persists in glia-restricted progenitors and differentiated glia [9,10]. The specification of DRG sensory neuron lineages is also controlled by several transcription factors. For example, the basic helix-loop-helix (bHLH) transcription factors Neurogenin-1 (*Ngn1*) and Neurogenin-2 (*Ngn2*) promote sensory fates, as opposed to autonomic ones [1,5,11,12], and are required for the initiation of neurogenesis [5]. *Neurogenic differentiation 1 *(*NeuroD1*) is thought to act downstream of the neurogenins in the regulation of neuronal differentiation [13,14]. As terminal differentiation progresses, sensory neuron subtypes with distinct modalities acquire specific patterns of Trk expression, uniquely expressing either TrkA, TrkB, or TrkC [1,15].

The function of Notch signaling in DRG development, as revealed by *in vitro *studies and *in vivo *chick studies, is consistent with its two fundamental roles in the development of the nervous system: maintaining undifferentiated progenitors by inhibiting neuronal differentiation, and promoting glial determination [16,17]. Studies of chick development show that Notch signaling is essential for the maintenance of NCCs and prevents neuronal differentiation in the DRG and sympathetic ganglion [18]. Furthermore, transient activation of Notch signaling in NCCs *in vitro *prevents neuronal differentiation and promotes glial differentiation [19,20]. On the other hand, results from mutant mice with Notch signaling pathway deletions are not consistent. *Delta-like-1 *(a Notch ligand) null mice had aberrantly fused and/or reduced DRG and sympathetic ganglia, suggesting that Notch signaling is also essential for the proper migration of NCCs [21]. *Hes1 *and *Hes5 *(Notch signaling effectors) double-null mutant mice maintain the expression of the specific marker for glial cell precursors, brain fatty acid binding protein (BFABP), in the DRG, suggesting that Notch signaling is not involved in the generation of glial cell precursors from NCCs [22,23]. By over-expressing or inactivating Notch signaling specifically in Schwann cell precursors *in vivo*, Notch signaling has been shown to drive the differentiation of immature Schwann cells [23]. Thus, manipulating individual components of Notch signaling in the mouse yields varying results and the full scope of Notch functions in mammalian DRG development remains to be elucidated.

The presence of four Notch receptors and at least five Notch ligands, all of which are partially functionally redundant, has made it difficult to investigate the physiological function of Notch signaling in mammals [16]. Recombination signal binding protein for immunoglobulin kappa J region (*Rbpj*) can interact with the intracellular domains of all four Notch receptors and is required to mediate their transcriptional effects [24,25]. Therefore, deletion of *Rbpj *would be expected to completely abolish canonical Notch signaling. Taylor *et al. *[26] conditionally knocked out *Rbpj *expression in NCCs and NCC-derived cells and observed a modest reduction in sensory neurons and profound defects in gliogenesis in the DRG of *Wnt1-Cre;Rbpj*^*flox/flox *^(*Rbpj *conditional knock-out (CKO)) mice. However, the whole range of DRG phenotypes, especially the defects of neurogenesis, in *Rbpj *CKO mice has not yet been described, and the mechanisms underlying the phenotype remain unclear.

In the present study, we focused on early developmental events in the DRG of *Rbpj *CKO mice. We found that *Ngn1 *and *Ngn2 *expression was unchanged in the absence of *Rbpj*, but *NeuroD1 *was up-regulated and precocious neurogenesis occurred in the DRG. The elevated rate of neuronal differentiation at early time points was followed by a reduction in cell proliferation and abnormal cell death. In addition, the initiation of BFABP expression in glial progenitors was delayed, and this expression was lost at later stages. In wild-type mice, P75 and Sox10 are co-expressed in NCCs during early DRG development, but are subsequently expressed in two distinct populations as development progresses [27]. In contrast, this separation did not occur in *Rbpj*-deficient DRG, as evidenced by the presence of P75/Sox10 co-expressing cells at later developmental stages, suggesting that the multipotency of NCCs was abnormally maintained, thus arresting their development. These data provide further insights into the physiological functions of Notch signaling in the development of DRG.

## Results

### Normal induction and early migration of NCCs in *Rbpj *CKO embryos

**Figure 1:**
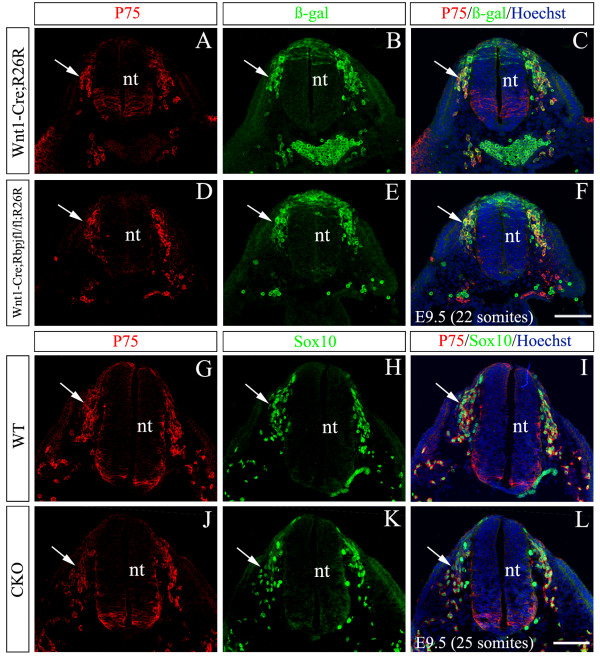
**Normal induction and early migration of NCCs in *Rbpj *CKO mice**. **(A-L) **Transverse sections through the upper neural tube (nt) at the 22 or 25 somite stage of E9.5 wild-type (WT) and *Rbpj *CKO mice with P75 (red) and β-gal (green) (A-F) or Sox10 (green) (G-L) antibodies and counterstained with Hoechst. Although Cre-mediated recombination has occurred in many P75^+ ^and Sox10^+ ^cells by this stage, loss of *Rbpj *does not appear to affect the expression of either protein. Arrows point to P75/β-gal (A-F) and P75/Sox10 (G-L) co-labeled migratory NCCs. Scale bars: 100 μm.

In order to determine at what time point *Rbpj *becomes inactivated in NCCs and their derivatives in *Rbpj *CKO mice, we performed X-gal staining of *Wnt1-Cre;Rosa26 *reporter (R) embryos that express β-galactosidase (β-gal) in the Cre expression domain. The X-gal product did not appear in the roof plate of the neural tube or in the presumptive DRG until E9.5 (data not shown). At this stage, nearly all P75^+ ^NCCs were also positively labeled with β-gal antibody in both *Rbpj *CKO (*Wnt1-Cre;Rbpj*^*flox/flox*^*;Rosa26R*) and control (*Wnt1-Cre;Rosa26R*) embryos (Figure 1A-F), demonstrating that *Rbpj *could be deleted in NCCs and their derivatives from E9.5 on. P75 and Sox10 are co-expressed in pre-migratory and migrating NCCs [27]. The double staining of P75 and Sox10 showed there was no obvious difference in the distribution and number of P75/Sox10-double labeled cells between CKO and wild-type embryos at E9.5 (Figure 1G-L). This result is consistent with a previous report [26] and suggests that deleting *Rbpj *from E9.5 does not affect the induction and initial migration of NCCs.

### Precocious neurogenesis in *Rbpj*-deficient DRG

**Figure 2:**
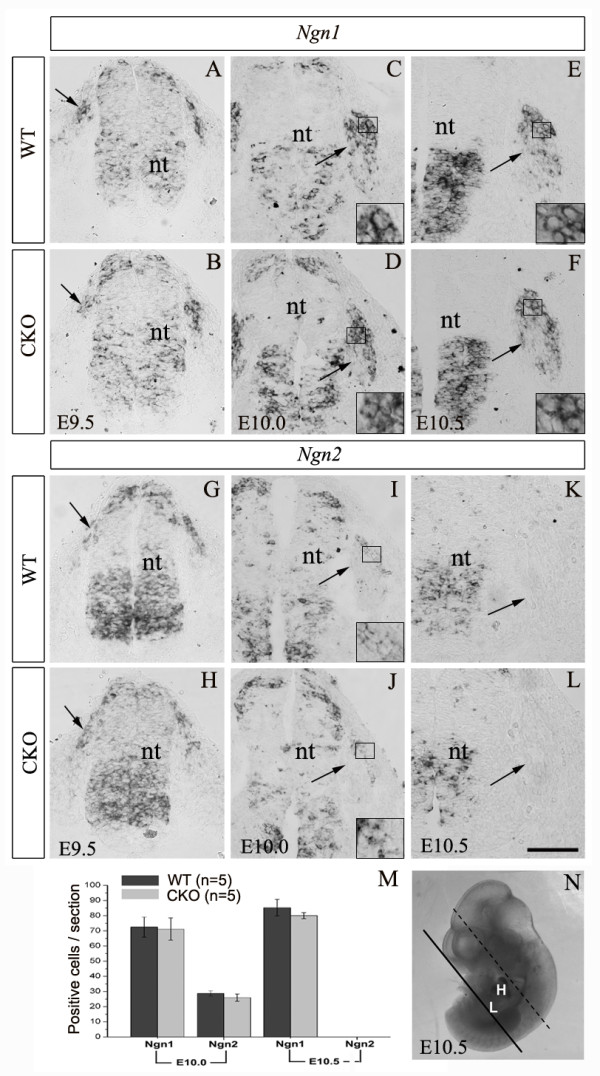
**Normal expression of neurogenins in *Rbpj*-deficient DRG**. **(A-L) **Transverse sections through the upper neural tube (nt) and surrounding tissue of wild-type (WT) and *Rbpj *CKO with *Ngn1 *(A-F) and *Ngn2 *(G-L) mRNA probes at the indicated stages. Loss of *Rbpj *does not appear to affect the expression of neurogenins either in migrating NCCs at E9.5 and E10.0, or in post-migratory NCCs in the DRG at E10.0 and E10.5. Arrows in (A,B,G,H) point to a cluster of migrating NCCs, and those in (C-F,I-L) point to post-migratory NCCs condensed in the DRG located laterally to the neural tube. High magnification views of the areas delineated by black rectangles in panels (C-F,I,J) are shown at the bottom of each panel. Note that the signal of *in situ *hybridization is present in the cytoplasm, whereas the nuclei contain no signals. (**M**) Comparison of the number of *Ngn1*^*+ *^and *Ngn2*^*+ *^cells in the DRG between wild-type and *Rbpj *CKO mice at E10.0 and E10.5. Error bars represent standard error of the mean. (**N**) Black line on representative E10.5 embryo indicates the level at which embryos was cut transversely. Upper part of embryos were placed vertically (relative to the spinal cord) on the frozen pedestal for sectioning. Cell counting from E10.0 to E11.5 embryos was performed in the thoracic DRGs between black and dashed black lines. H, heart; L, liver; nt, neural tube. Scale bars: 100 μm.

We next examined neurogenesis in *Rbpj*-deficient DRG by looking at the expression of neurogenins in these mice. *Ngn1 *and *Ngn2 *are required for the specification of DRG sensory neurons [5]. In E9.5 wild-type embryos, *Ngn1 *and *Ngn2 *were expressed in a cluster of migratory NCCs (Figure 2A,G). The expression domain of *Ngn1 *was enlarged at E10.0 and E10.5 (Figure 2C,E), whereas *Ngn2 *expression was unchanged at E10.0 and had disappeared by E10.5 (Figure 2I,K), a stage at which differentiating sensory neurons are condensed into ganglia [28]. We did not observe any obvious difference in *Ngn1 *and *Ngn2 *expression between wild-type and CKO embryos from E9.5 to E10.5 (Figure 2), suggesting that the specification of sensory neurons may not be affected in the absence of *Rbpj*.

**Figure 3:**
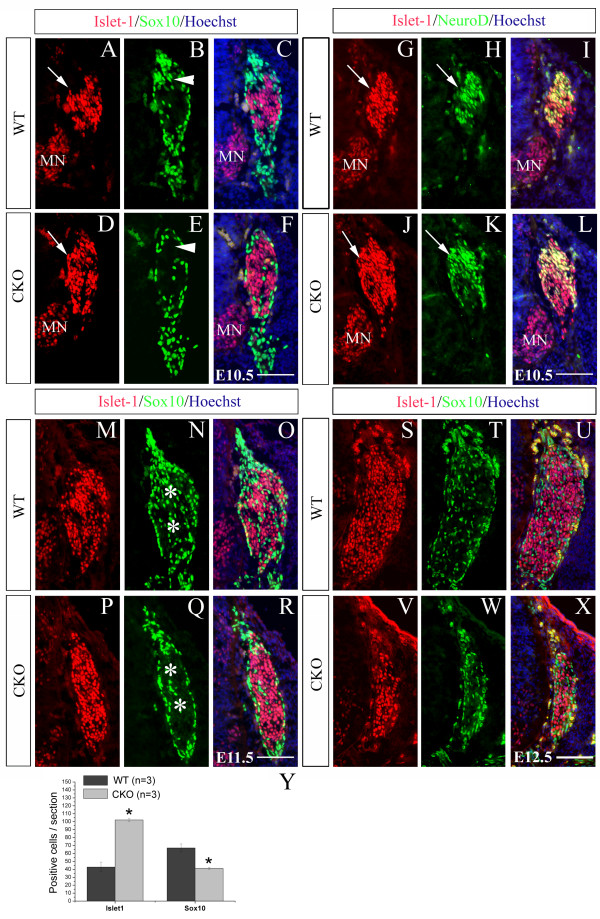
**Precocious neurogenesis and loss of sensory neurons in *Rbpj*-deficient DRG**. **(A-X) **Transverse sections through the DRG of wild-type (WT) and *Rbpj *CKO mice for the indicated markers and counterstained with Hoechst at E10.5 (A-L), E11.5 (M-R) or E12.5 (S-X). At E10.5, Islet1/Sox10 double staining reveals an increase in the number of Islet1^+ ^cells, but a decrease in that of Sox10^+ ^cells in *Rbpj*-deficient DRG compared with wild-type controls (A-F). Note that many Islet1-expressing cells (arrows in A,D) are abnormally distributed in the upper portion of the *Rbpj *CKO DRG, a region normally occupied by Sox10^+ ^cells (arrowheads in B,E) in wild-type DRG. At E11.5 (M-R), Sox10^+ ^cells are distributed throughout wild-type DRG (asterisks in N), but are almost absent in the center of *Rbpj*-deficient DRG (asterisks in Q). At E12.5 (S-X), numbers of both Islet1^+ ^and Sox10^+ ^cells are reduced in *Rbpj*-deficient DRG compared with wild-type. (G-L) Double staining of Islet1 and NeuroD1 shows an increase in the number of NeuroD1^+ ^cells in *Rbpj*-deficient DRG relative to wild-type controls at E10.5. Note that NeuroD1 and Islet are co-expressed in sensory neurons. Arrows in (G-K) point to the DRG. MN, motor neuron. Scale bars: 100 μm. (**Y) **Comparison of the number of Islet1^+ ^or Sox10^+ ^cells at the DRG between wild-type and *Rbpj *CKO mice at E10.5. Error bars represent standard error of the mean; *P < 0.01.

**Figure 4:**
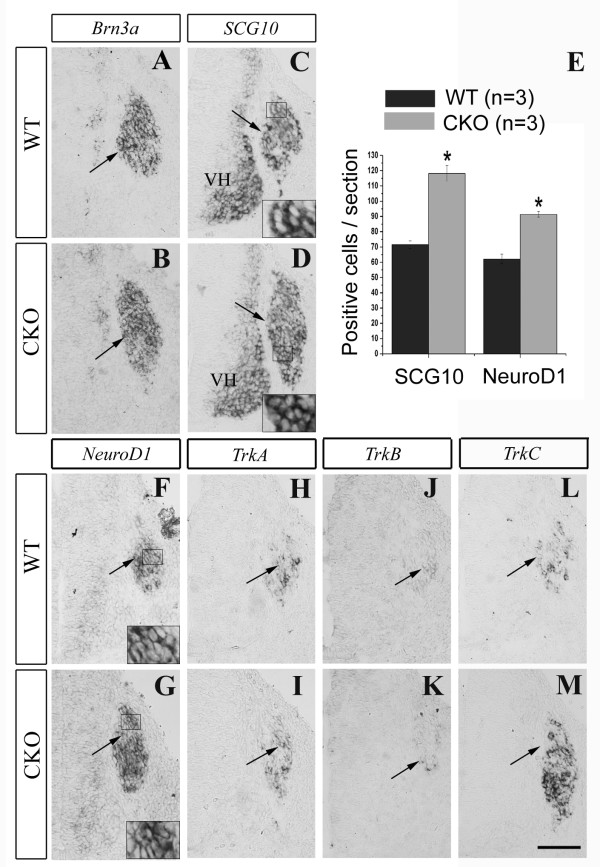
**Increased *TrkC***^**+ **^**neurons in *Rbpj*-deficient DRG at E10.5**. **(A-D,F,G) **Transverse sections through DRG of wild-type (WT) and *Rbpj *CKO hybridized with the indicated probes. *Brn3a *(A,B), *SCG10 *(C,D) and *NeuroD1 *(F,G) expression levels are all enhanced in *Rbpj*-deficient DRG compared with wild-type controls. High magnification views of the areas delineated by black rectangles in (C,D,F,G) are shown at the bottom of each panel. **(E) **Comparison of the number of *SCG10*^+ ^or *NeuroD1*^+ ^cells in the DRG between wild-type and *Rbpj *CKO mice at E10.5. Error bars represent standard error of the mean; *P < 0.05. **(H-M) **The expression of *TrkA *(H,I) and *TrkB *(J,K) does not differ notably between the two groups, but *TrkC *expression (L,M) is greatly up-regulated in *Rbpj*-deficient DRG compared with wild-type controls. Arrows point to the DRG. VH, ventral horn. Scale bars: 100 μm.

Detectable defects in neurogenesis in *Rbpj *CKO mice were first observed at E10.5, a stage when nearly all primary sensory neurons co-express the POU homeodomain transcription factor Brn3a and the LIM homeodomain transcription factor Islet1 [29], and the onset of their expression coincides largely with the commitment of NCCs to neuronal fates [28]. We found that the numbers of both Islet1^+ ^(Figure 3A,D,Y) and *Brn3a*^+ ^(Figure 4A,B) cells were dramatically increased and that of Sox10^+ ^cells (Figure 3B,E,Y) was decreased in *Rbpj *CKO mice at E10.5 compared with wild types. Furthermore, many Islet1-expressing cells were abnormally distributed in the upper portion of the DRG in CKO embryos (arrows in Figure 3A,D), an area normally occupied by Sox10^+ ^cells in the wild-type DRG (arrowheads in Figure 3B,E). The overall increase in the number of neurons in the E10.5 CKO DRG was confirmed by increased expression of the pan-neuronal marker *SCG10 *(Figure 4C,D,E). To explore the mechanisms underlying precocious neurogenesis in the CKO DRG, we examined the expression of the proneural gene *NeuroD1*. *In situ *hybridization showed a great increase in *NeuroD1 *expression in the DRG of CKO embryos relative to wild-type controls at E10.5 (Figure 4E,F,G), and co-immunostaining revealed that almost all NeuroD1^+ ^neurons were also positive for Islet1 (Figure 3G-L). Taken together, our data indicate that, in the absence of *Rbpj*, DRG progenitors precociously differentiate into sensory neurons, possibly due to up-regulation of *NeuroD1*.

### Reduced cell proliferation and abnormal cell death in *Rbpj*-deficient DRG

**Figure 5:**
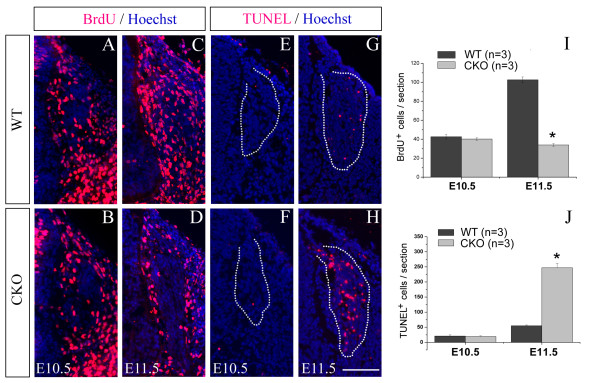
**Inactivation of *Rbpj *results in reduced cell proliferation and increased apoptosis in E11.5 DRG **. Wild-type (WT) and *Rbpj *CKO embryos were pulse labeled with BrdU 2 hours prior to fixation. **(A-D) **BrdU incorporation in *Rbpj*-deficient DRG is comparable to that in wild-type controls at E10.5 (A,B), but greatly reduced at E11.5 (C,D). **(E-F) **TUNEL staining of transverse sections through DRG (outlined) shows that there is no difference in the rate of apoptosis between wild-type and CKO DRG at E10.5 (E,F), but the number of TUNEL-labeled cells is significantly increased in *Rbpj*-deficient DRG relative to controls at E11.5 (G,H). Scale bar: 100 μm. **(I,J) **Statistical analysis of BrdU^+ ^(I) or TUNEL^+ ^cells (J) in wild-type and *Rbpj *CKO DRG. Error bars represent standard error of the mean; *P < 0.01.

The increase in the number of sensory neurons in the DRG of *Rbpj *CKO mice was not obvious at E11.5 (Figure 3M-R), and by E12.5 the number was dramatically reduced relative to wild-type littermates (Figure 3S-X), consistent with a previous report [26]. We reasoned that the precocious neurogenesis caused by the deletion of *Rbpj *led to premature depletion of the progenitor pool, which in turn resulted in an overall reduction in sensory neuron production. To test this possibility, we pulse labeled the E10.5 to E11.5 DRG with bromodeoxyuridine (BrdU) and analyzed the rates of proliferation of progenitor cells 2 hours later. There was no significant difference in BrdU incorporation between wild-type and CKO mice at E10.5, but the number of BrdU-labeled cells was greatly reduced in the CKO DRG at E11.5 (Figure 5A-D,I).

**Figure 6:**
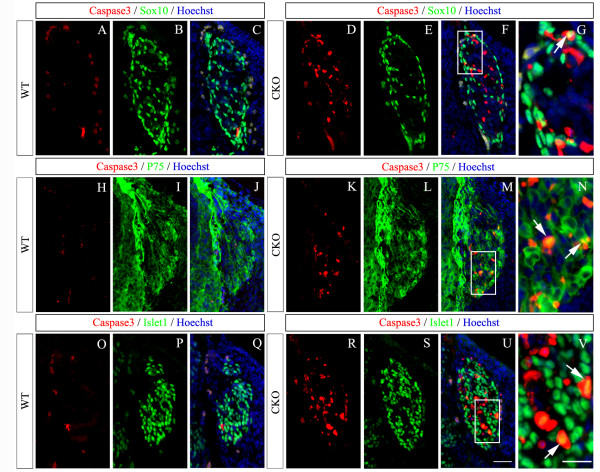
**Apoptosis occurs both in precursor cells and early differentiating neurons in *Rbpj *CKO DRG at E11.5**. **(A-V) **Double immunostaining of Caspase3 with Sox10 (A-G), P75 (H-N) and Islet1 (O-V). There are few Caspase^+ ^cells in wild-type (WT) DRG (A,H,O), whereas many Caspase^+ ^cells are present in CKO DRG (D,K,R). Some Caspase^+ ^cells are immunostained with Sox10 (D-G), P75 (K-N) and Islet1(R-V) in CKO DRG. (G,N,V) High magnification views of the areas delineated by white rectangles in (F,M,U), respectively. Arrows indicate the double-labeled cells. Scale bars: 100 μm in (A-F,H-M,O-U); 25 μm in (G,N,V).

To determine whether elevated rates of cell death may also have contributed to the overall reduction of sensory neurons, we performed terminal deoxynucleotidyl transferase dUTP nick end labeling (TUNEL) staining. No difference in TUNEL staining between the wild-type and CKO DRG was observed at E10.5, but the number of TUNEL^+ ^cells was increased in CKO mice relative to controls at E11.5 (Figure 5E-H,J). In order to explore which kinds of cells are dying in the E11.5 DRG of CKO mice, Caspase3, an apoptosis marker, was co-immunostained with Sox10 (Figure 6A-G), P75 (Figure 6H-N) or Islet1 (Figure 6X-V). Like TUNEL staining, many Caspase3^+ ^cells were present in CKO mice, whereas they were rarely observed in wild-type controls at E11.5 (Figure 6). Co-immunostaining showed Caspase3 was co-localized with Sox10 (Figure 6G), P75 (Figure 6N) or Islet1 (Figure 6V), showing that abnormal cell death occurs in both progenitor cells and early differentiating neurons. Taken together, these results suggest that reduced cell proliferation and increased cell death contribute to the reduction of sensory neurons in *Rbpj *CKO mice.

**Figure 7:**
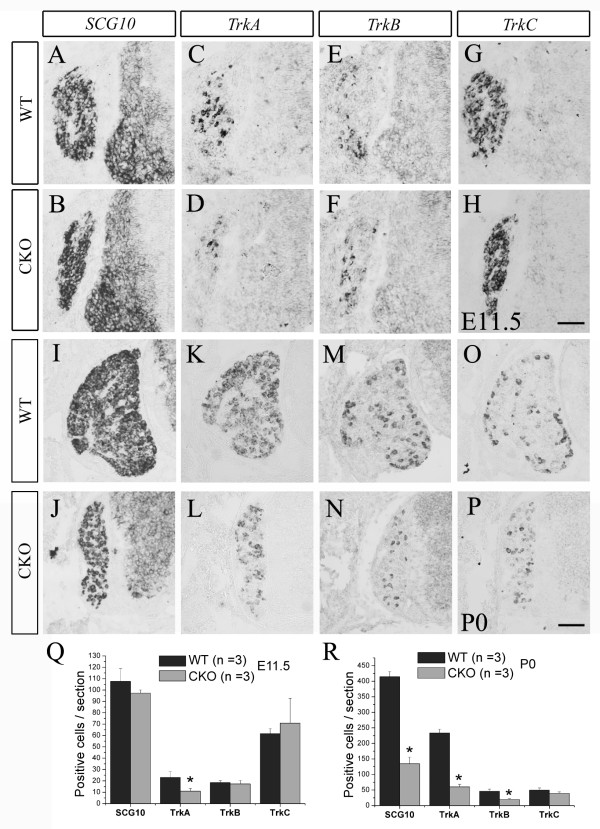
**Reduced numbers of *TrkA***^***+***^**and *TrkB***^***+***^**neurons in *Rbpj*-deficient DRG**. **(A-P) ***SCG10 *(A,B,I,J), *TrkA *(C,D,K,L), *TrkB *(E,F,M,N) and *TrkC *(G,H,O,P) *in situ *hybridization of transverse sections through DRG of wild-type (WT) and *Rbpj *CKO mice at E11.5 and post natal day (P)0. Scale bars: 100 μm. **(Q) **At E11.5, *TrkA*^+ ^neurons are significantly decreased in *Rbpj *CKO DRG and there are no significant differences in the number of *SCG10*^*+*^, *TrkB*^*+ *^and *TrkC*^*+ *^neurons between wild-type and *Rbpj *CKO mice. **(R) **The numbers of *SCG10*^*+*^, *TrkA*^*+ *^and *TrkB*^*+ *^neurons are significantly decreased, but that of *TrkC*^*+ *^neurons is unchanged in *Rbpj*-deficient DRG relative to wild-type controls at P0. Error bars represent standard error of the mean; *P < 0.01.

As mentioned above, sensory neurons in the DRG can be divided into three groups based on their expression of the three *Trk *genes [1]. To determine whether *Rbpj *deletion has distinct effects on the development of different types of sensory neurons, we examined the expression of *TrkA*, *TrkB *and *TrkC*. We found that the number of *TrkC*^*+ *^neurons in *Rpbj *CKO DRG was greatly increased (Figure 4 L,M), but the numbers of *TrkA*^*+ *^and *TrkB*^*+ *^neurons were not changed at E10.5 (Figure 4H-K). The significant increase of *TrkC*^*+ *^neurons in *Rpbj *CKO DRG no longer existed at E11.5 (Figure 7G,H,Q), and *TrkA*^*+ *^neurons in *Rpbj *CKO DRG were reduced in number compared with wild-type mice (Figure 7C,D,Q). At postnatal day 0, the number of *TrkC*^*+ *^neurons in *Rpbj *CKO DRG was comparable to that of wild-type mice (Figure 7O,P,R), but *TrkA*^*+ *^and *TrkB*^*+ *^neurons were greatly reduced in CKO mice (Figure 7K-N,R). During DRG morphogenesis, large *TrkC*^+ ^neurons are the first to be generated [1]; thus, the selective increase in *TrkC*^+ ^neurons supports the idea that deletion of *Rbpj *leads to enhanced neurogenesis during the first wave of NCC differentiation, but this results in a depletion of the neural progenitor pool, which in turn leads to the reduction of consequent generation of *TrkA*^+ ^and *TrkB*^+ ^neurons. Of course, abnormal cell death also contributes to the reduction of these two types of sensory neurons.

### Severe gliogenesis defects in *Rbpj*-deficient DRG

**Figure 8:**
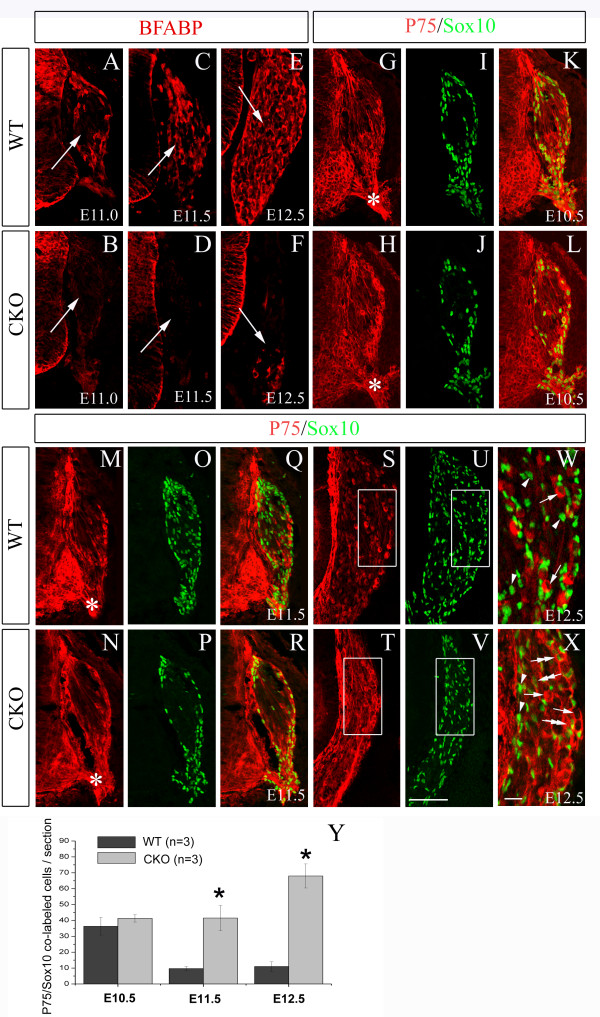
**Inactivation of *Rbpj *results in severe gliogenesis defects**. **(A-F) **BFABP immunostaining of wild-type (WT) and *Rbpj *CKO DRG at E11.0 (A,B), E11.5 (C,D) and E12.5 (E,F). Compared with wild type controls, BFABP expression in *Rbpj*-deficient DRG is undetectable at E11.0 (B) and E11.5 (D), and only a small number of labeled cells are observed at E12.5 (F). Arrows point to DRGs. **(G-X) **Double immunolabeling of P75 and Sox10 at E10.5 (G-L), E11.5 (M-R) and E12.5 (S-X). P75/Sox10 co-labeled cells are observed in both wild-type and CKO DRGs at E10.5 (G-L), and there were no obvious differences in the number of co-labeled cells between wild-type and CKO. In wild-type DRG at E11.5 (Q) and E12.5 (W), P75 and Sox10 are distinctly expressed in separate populations of cells, whereas many P75/Sox10 co-labeled cells are present in CKO DRG at these stages (R,X). (W,X) High magnification views of the areas delineated by white rectangles in (S,U) and (T,V), respectively. Arrows indicate cells that express P75 alone, arrowheads indicate cells that express Sox10 alone, and double arrows indicate P75/Sox10 co-labeled cells. **(Y) **Statistical analysis of numbers of P75/Sox10 co-labeled cells in wild-type and *Rbpj *CKO DRG at E10.5, E11.5 and E12.5. Error bars represent standard error of the mean; *P < 0.05. Note that P75/Sox10 co-labeled cells located at the nerve root (asterisks in G,H,M,N) were not counted. Scale bars: 100 μm in (A-V); 20 μm in (W,X).

Taylor *et al. *[26] showed that severe gliogenesis defects are present in *Rbpj*-deficient DRG. Consistent with their findings, we also found a complete loss of BFABP (a glia-specific marker) expression in the *Rbpj*-deficient DRG at E16.5 (Additional file 1). At E11.0, a half day after the occurrence of precocious neurogenesis in *Rbpj *CKO mice, BFABP expression is initiated in glia-restricted progenitors of the wild-type DRG (Figure 8A) [30]. In contrast, BFABP expression was not detected in the DRG of *Rbpj *CKO mice at this stage (Figure 8B). By E12.5, only a few BFABP-labeled cells were observed in *Rbpj *CKO ganglia, whereas many BFABP^+ ^cells were distributed throughout wild-type DRG (Figure 8E,F). Thus, BFABP expression in *Rbpj *CKO DRG was delayed and drastically reduced as development progressed.

P75 and Sox10 are co-expressed in multipotent NCCs at E8.5 to E9.5, but these genes gradually become expressed in distinct DRG progenitor pools as the DRG condenses; after E10.5, P75-expressing cells commit to the neuronal lineage while Sox10-expressing cells become glia [27]. To explore the mechanism underlying defective gliogenesis, we performed double immunolabeling of P75 and Sox10 in CKO mice. At E10.5, there was no apparent difference in P75 expression between wild-type and *Rbpj *CKO DRG, and similar numbers of P75/Sox10 co-labeled cells were observed in both genotypes (Figure 8G-L,Y). At E11.5 and E12.5, few P75/Sox10 co-labeled cells were observed in wild-type mice, revealing the segregation of P75 and Sox10 expression that occurs as DRG development progresses (Figure 8M,O,Q,W,Y). In contrast, P75 expression was increased in *Rbpj *CKO mice at E11.5 and E12.5, and many P75/Sox10 co-labeled cells were present, particularly at the DRG periphery (Figure 8N,P,R,X,Y). Note that P75 and Sox10 localize to the cytoplasm and nucleus, respectively, and co-labeled cells are easily identified when imaged at high magnification (Figure 8W,X). The presence of P75/Sox10-co-labeled cells suggests that the restriction and bifurcation of NCC fates from E10.5 fails to occur in *Rbpj *CKO DRG.

## Discussion

In the present study, we specifically inactivated the critical transcription factor downstream of all four Notch receptors, Rbpj, in NCCs and their derivatives. Up-regulation of NeuroD1 and precocious neurogenesis were observed in the DRG of *Rbpj *CKO mice, followed by reduced proliferation and abnormal cell death. These phenotypes were not reported in a previous examination of *Rbpj *CKO (*Wnt1-Cre;Rbpj*^*flox/flox*^) mice [26]. We confirmed previous findings revealing a near-complete loss of glia in *Rbpj*-deficient DRG [26], and further found that a large number of P75/Sox10 co-expressing NCCs were abnormally maintained in *Rbpj *CKO mice, suggesting that defective NCC development contributes to the loss of glia in these mice.

### The role of *Rbpj *in DRG neurogenesis

We observed precocious neurogenesis in *Rbpj-*deficient DRG, a phenotype consistent with the known role of canonical Notch signaling in maintaining the undifferentiated state of neural progenitors by inhibiting the expression of genes involved in neuronal differentiation [16]. This finding is at odds with those of a recent study that reported there was no evidence of premature neuronal differentiation in *Rbpj*-deficient DRG, a conclusion based on Tuj1 and Peripherin expression patterns [26]. In the present study, we analyzed Islet1 and Brn3a expression to determine the timing and extent of neurogenesis in the DRG, and found that an elevated number of cells expressed these sensory neuronal markers between E10.5 and E11.5. Consistent with these observations, we found that BrdU incorporation in *Rbpj*-deficient DRG was less prevalent at this stage of development, indicating that NCC progenitor cells were prematurely differentiating into neurons. After E11.5, however, Islet1 and Brn3a staining suggested that the number of sensory neurons in *Rbpj*-deficient DRG was reduced. Therefore, we conclude that arresting Notch signaling in DRG NCCs removes critical inhibition, allowing them to differentiate into neurons at an exuberant rate during the early stages of DRG development, leading to depletion of the progenitor pool and an overall deficit of sensory neurons. Furthermore, the sensory neuron deficit in *Rbpj*-deficient DRG from E12.5 was also partly due to increased apoptosis, which may have occurred as a consequence of the uncoordinated development of the DRG.

To explore the mechanism underlying precocious neurogenesis, we examined the expression of several genes involved in the specification and differentiation of sensory neurons. Previous studies have shown that Notch signaling acts through the *Hes *genes, transcriptional co-repressors activated by Notch signaling that inhibit the expression of proneural genes, such as *Ngn1 *and *Ngn2*. This repression, in turn, prevents the activation of neurogenic differentiation genes such as *NeuroD *[17,31]. However, we detected no difference in the expression of *Ngn1 *and *Ngn2 *in the DRG between *Rbpj *CKO and wild-type mice, but observed a great up-regulation of *NeuroD1 *in *Rbpj*-deficient DRG. These results suggest that *Rbpj *normally inhibits neuronal differentiation of NCCs in the DRG by repressing *NeuroD1 *expression via a novel mechanism, which is independent of *Ngn1 *and *Ngn2*. On the other hand, evidence from studies of retina development shows that *NeuroD1 *governs the neuron versus glial fate decision by promoting neurogenesis and suppressing gliogensis [32,33], and it is unclear whether up-regulation of *NeuroD1 *is also involved in defective gliogenesis in CKO mice (see below).

### Mechanisms underlying severe gliogenesis defects in *Rbpj *CKO mice

Premature neuronal differentiation, reduced cell proliferation, and abnormal cell death in progenitor cells were all observed in *Rbpj*-deficient DRG. However, these phenotypes cannot fully account for the severity of the observed defects in gliogenesis. Consistent with previous findings [26], we found that the initiation of glia-specific expression of BFABP was delayed in *Rbpj *CKO mice, and expression in the relatively small population of cells was lost at later developmental stages. In wild-type NCCs, P75 and Sox10 are co-expressed early in DRG development, but as development progresses P75 becomes restricted to neuronal precursors while Sox10 becomes restricted to glial precursors [27]. Interestingly, we found that this separation did not occur in *Rbpj*-deficient NCCs, and P75/Sox10 co-expressing cells were still observed at E12.5. These results suggest that, in the absence of *Rbpj*, the subpopulation of NCCs that normally become restricted to glial fates fails to differentiate and instead maintains pluripotency, thus retaining the potential to differentiate into neurons. Taylor *et al. *[26] showed that these NCCs also maintain glial fate potential and can be induced to differentiate normally *in vitro *by stimulation with the gliogenic factor Neuregulin1-β1. Furthermore, BFABP is known to play a critical role in gliogenesis [34,35], Interestingly, the *BFABP *promoter contains an *Rbpj *binding site that is essential for *BFABP *transcription in radial glial cells [36], and thus it is likely that *Rbpj *promotes glial differentiation in the DRG by directly activating the transcription of *BFABP*.

The reduction in Sox10 expression was one of the earliest defects that we observed in *Rbpj *CKO mice, suggesting that Notch signaling is necessary for the maintenance of Sox10 expression. Because Sox10 maintains multipotency, inhibits neuronal differentiation of NCCs, and serves as a key regulator for peripheral glial development [9,37], we speculated that the reduction in Sox10 expression might contribute to defects in both gliogenesis and neurogenesis. Consistently, DRG phenotypes observed in *Sox10 *null mice [27,37] are very similar to those of *Rbpj *CKO mice. In addition, a study on the enteric nervous system proposed that, by suppressing proneural genes such as *Mash1*, Notch signaling might be required for continuous *Sox10 *expression and the maintenance of enteric neural crest progenitors [38]. In light of the up-regulation of *NeuroD1 *and the reduction of *Sox10*, we propose that *Rbpj *might maintain *Sox10 *expression by repressing *NeuroD1*. However, further studies are required to determine the relationships among them, as well as their combined effects on differentiation processes in precursor cells of the DRG.

## Conclusions

We conditionally knocked out the transcription factor *Rbpj*, a master integrator of activation signals from all Notch receptors, in NCCs, and examined the role of *Rbpj *in early events of DRG development. Premature neuronal differentiation, reduced cell proliferation, and increased apoptosis in both progenitor cells and early differentiating neurons were all observed in *Rbpj*-deficient DRG. The up-regulation of *NeuroD1 *in the absence of *Rbpj *may lead to premature neuronal differentiation, and abnormal maintenance of stem cell potential by NCCs may contribute to the profound defects in gliogenesis as well as in neurogenesis in *Rbpj *CKO mice.

## Materials and methods

### Mouse breeding and genotyping

*Wnt1-Cre*, *Rbpj*^*flox/flox *^and Rosa26 reporter mice were generated and genotyped as previously described [39-41]. To inactivate *Rbpj *expression in the neural crest, we crossed *Wnt1*-Cre mice with *Rbpj*^*flox/flox *^mice to obtain *Wnt1-Cre;Rbp*^*flox/+ *^progeny. Then *Wnt1-Cre;Rbp*^*flox/+ *^mice were crossed with each other to obtain *Wnt1-Cre; Rbp*^*flox/flox *^progeny. The morning of the day on which the vaginal plug appeared was designated as E0.5. In each set of experiments, at least three CKO embryos or pups in each experimental group and an equal number of littermate control mice (for example, wild-type, *Rbpj*^*flox/+ *^or *Wnt1-Cre;Rbpj*^*flox/+*^) were used. Animal care procedures were reviewed and approved by the Animal Studies Committee at the Tongji University School of Medicine, Shanghai, China.

### *In situ *hybridization and immunocytochemistry

*In situ *hybridization and immunocytochemistry on brain sections were performed as previously described [42]. The following mouse antisense RNA probes were used: *Ngn1*, *Ngn2 *[14], *NeuroD1 *[43], *SCG10*, *TrkA*, *TrkB*, and *TrkC *[5]. The probe *Brn3a *(NM_0011143; 0.60 kb) was generated by PCR using cDNA templates prepared from E10.5 mouse embryos. For immunohistochemistry, the following primary antibodies were used: goat anti-β-gal (1:1,000; AbD Serotec, Kidlington, OX5 1GE, UK), rabbit anti-P75 (1:500; Promega, Fitchburg, Wisconsin, USA), goat anti-Sox10 (1:500; Santa Cruz, California, USA ), mouse anti-Islet1 (1:100; Developmental Studies Hybridoma Bank, Iowa, USA), rabbit anti-BFABP (1:1,000; Chemicon, California, USA), goat anti-NeuroD1(1:200; Santa Cruz), mouse anti-BrdU (1:200; Calbiochem, Darmstadt, Germany), rabbit anti-β-tubulin III (1:1,000; Sigma, St. Louis, USA), rabbit anti-Caspase3 (1:1,000; Cell Signaling Technology, Boston, USA). Because anti-Caspase3 and anti-P75 antibodies were raised in rabbit, we used the tyramide signal amplification system (TSA cyanine 3 system; Perkin Elmer Life Sciences, Boston, MA, USA) to do double immunostaining [44]. Species-specific secondary antibodies conjugated to Cy2 or Cy3 (1:1,000; Jackson ImmunoResearch, West Grove, PA, USA) were used to detect primary antibodies. Sections were observed under a Nikon BOi or a Zeiss LSM510 confocal microscope.

### BrdU labeling and TUNEL staining

For BrdU labeling, a single BrdU pulse (60 μg/g of body weight) was delivered intraperitoneally to timed-pregnant females at E10.5 and E11.5, and embryos were fixed 2 hours later. Sections were processed for BrdU and BFABP or Sox10 double immunostaining as described above. TUNEL staining was performed according to the In Situ Cell Death Detection Kit instructions (Roche, Indianapolis, USA).

### Statistical analysis

For cell counts in E10.0, E10.5 or E11.5 DRG, we selected embryos with 26 to 28 somites as E10.0, and those with 36 to 38 somites as E10.5 [45]. In order to obtain transverse sections with similar angles, wild-type and mutant embryos at the desired stages with similar size were cut along the black line as shown in Figure 2N, and then the upper parts of embryos were positioned vertically (relative to the spinal neural tube) on the frozen pedestal prior to sectioning. Because DRG development shows big differences along the anteroposterior axis, counting data for comparison were collected from the sections at the thoracic level, which was determined by the appearance of the heart and liver. Six sets of consecutive 10-μm thick sections were made in each embryo, and cell counts were done in one set of sections that had been processed for *in situ *hybridization or immunostaining (see above). Approximately five sections on one slide were counted. Similarly, cell counts in post natal day 0 DRG were done on one set of consecutive 20-μm thick cryostat sections through lumbar DRGs. Cell counting was conducted by a co-author who did not know the genotyping data. For each set of comparisons, at least three CKO mice and three littermate controls (for example, wild-type, *Rbpj*^*flox/+ *^or *Wnt1-Cre;Rbpj*^*flox/+*^) were included. All data were analyzed using OriginPro7.5 [46] software and are presented as mean ± standard error of the mean. Comparisons were made using an unpaired Student's *t*-test and statistical significance was set at *P *< 0.05.

## Abbreviations

β-gal: β-galactosidase; BFABP: brain fatty acid binding protein; BrdU: bromodeoxyuridine; CKO: conditional knock-out; DRG: dorsal root ganglion; E: embryonic day; NCC: neural crest stem cell; Trk: tyrosine receptor kinase; TUNEL: terminal deoxynucleotidyl transferase dUTP nick end labeling.

## Competing interests

The authors declare that they have no competing interests.

## Authors' contributions

ZLH and MS participated in the staining and counting procedures, YH, ZP and JYC worked on mouse breeding and genotyping, MHZ and HH provided the *Rbpj*^*flox/flox *^mice and conceptually revised the manuscript, and YQD and ZLH designed the study and collaborated in the writing of the manuscript. All authors read and approved the final manuscript.

## Supplementary Material

Additional file 1 **Reduced number of neurons and near-complete loss of glia in *Rbpj*-deficient DRG at E16.5**. **(A-D) **Tuj1 (A,B) and BFABP (C,D) immunostaining of transverse sections through wild-type and *Rbpj*-deficient DRG at E16.5 with Hoechst counterstaining. Note that BFABP expression is present in the spinal cord, but not in the DRG (arrow) of *Rbpj *CKO mice. VH, spinal ventral horn. Scale bars: 100 μm. Click here for file

## References

[B1] Marmigere F Ernfors P Specification and connectivity of neuronal subtypes in the sensory lineage Nat Rev Neurosci 2007 8 114 127 10.1038/nrn2057 17237804

[B2] Le Douarin NM Creuzet S Couly G Dupin E Neural crest cell plasticity and its limits Development 2004 131 4637 4650 10.1242/dev.01350 15358668

[B3] Serbedzija GN Fraser SE Bronner-Fraser M Pathways of trunk neural crest cell migration in the mouse embryo as revealed by vital dye labelling Development 1990 108 605 612 2387238 10.1242/dev.108.4.605

[B4] Frank E Sanes JR Lineage of neurons and glia in chick dorsal root ganglia: analysis *in vivo *with a recombinant retrovirus Development 1991 111 895 908 1908772 10.1242/dev.111.4.895

[B5] Ma Q Fode C Guillemot F Anderson DJ Neurogenin1 and neurogenin2 control two distinct waves of neurogenesis in developing dorsal root ganglia Genes Dev 1999 13 1717 1728 10.1101/gad.13.13.1717 10398684 PMC316844

[B6] Maro GS Vermeren M Voiculescu O Melton L Cohen J Charnay P Topilko P Neural crest boundary cap cells constitute a source of neuronal and glial cells of the PNS Nat Neurosci 2004 7 930 938 10.1038/nn1299 15322547

[B7] Stemple DL Anderson DJ Isolation of a stem cell for neurons and glia from the mammalian neural crest Cell 1992 71 973 985 10.1016/0092-8674(92)90393-Q 1458542

[B8] Hapner SJ Boeshore KL Large TH Lefcort F Neural differentiation promoted by truncated trkC receptors in collaboration with p75(NTR) Dev Biol 1998 201 90 100 10.1006/dbio.1998.8970 9733576

[B9] Kim J Lo L Dormand E Anderson DJ SOX10 maintains multipotency and inhibits neuronal differentiation of neural crest stem cells Neuron 2003 38 17 31 10.1016/S0896-6273(03)00163-6 12691661

[B10] Kelsh RN Sorting out Sox10 functions in neural crest development Bioessays 2006 28 788 798 10.1002/bies.20445 16927299

[B11] Perez SE Rebelo S Anderson DJ Early specification of sensory neuron fate revealed by expression and function of neurogenins in the chick embryo Development 1999 126 1715 1728 10079233 10.1242/dev.126.8.1715

[B12] Zirlinger M Lo L McMahon J McMahon AP Anderson DJ Transient expression of the bHLH factor neurogenin-2 marks a subpopulation of neural crest cells biased for a sensory but not a neuronal fate Proc Natl Acad Sci USA 2002 99 8084 8089 10.1073/pnas.122231199 12060754 PMC123024

[B13] Ma Q Kintner C Anderson DJ Identification of neurogenin, a vertebrate neuronal determination gene Cell 1996 87 43 52 10.1016/S0092-8674(00)81321-5 8858147

[B14] Sommer L Ma Q Anderson DJ Neurogenins, a novel family of atonal-related bHLH transcription factors, are putative mammalian neuronal determination genes that reveal progenitor cell heterogeneity in the developing CNS and PNS Mol Cell Neurosci 1996 8 221 241 10.1006/mcne.1996.0060 9000438

[B15] Sun Y Dykes IM Liang X Eng SR Evans SM Turner EE A central role for Islet1 in sensory neuron development linking sensory and spinal gene regulatory programs Nat Neurosci 2008 11 1283 1293 10.1038/nn.2209 18849985 PMC2605652

[B16] Louvi A Artavanis-Tsakonas S Notch signalling in vertebrate neural development Nat Rev Neurosci 2006 7 93 102 10.1038/nrn1847 16429119

[B17] Cornell RA Eisen JS Notch in the pathway: the roles of Notch signaling in neural crest development Semin Cell Dev Biol 2005 16 663 672 10.1016/j.semcdb.2005.06.009 16054851

[B18] Tsarovina K Schellenberger J Schneider C Rohrer H Progenitor cell maintenance and neurogenesis in sympathetic ganglia involves Notch signaling Mol Cell Neurosci 2008 37 20 31 10.1016/j.mcn.2007.08.010 17920293

[B19] Morrison SJ Perez SE Qiao Z Verdi JM Hicks C Weinmaster G Anderson DJ Transient Notch activation initiates an irreversible switch from neurogenesis to gliogenesis by neural crest stem cells Cell 2000 101 499 510 10.1016/S0092-8674(00)80860-0 10850492

[B20] Wakamatsu Y Maynard TM Weston JA Fate determination of neural crest cells by NOTCH-mediated lateral inhibition and asymmetrical cell division during gangliogenesis Development 2000 127 2811 2821 10851127 10.1242/dev.127.13.2811

[B21] De Bellard ME Ching W Gossler A Bronner-Fraser M Disruption of segmental neural crest migration and ephrin expression in delta-1 null mice Dev Biol 2002 249 121 130 10.1006/dbio.2002.0756 12217323

[B22] Hatakeyama J Sakamoto S Kageyama R Hes1 and Hes5 regulate the development of the cranial and spinal nerve systems Dev Neurosci 2006 28 92 101 10.1159/000090756 16508307

[B23] Woodhoo A Alonso MB Droggiti A Turmaine M D'Antonio M Parkinson DB Wilton DK Al-Shawi R Simons P Shen J Guillemot F Radtke F Meijer D Feltri ML Wrabetz L Mirsky R Jessen KR Notch controls embryonic Schwann cell differentiation, postnatal myelination and adult plasticity Nat Neurosci 2009 12 839 847 10.1038/nn.2323 19525946 PMC2782951

[B24] Kato H Sakai T Tamura K Minoguchi S Shirayoshi Y Hamada Y Tsujimoto Y Honjo T Functional conservation of mouse Notch receptor family members FEBS Lett 1996 395 221 224 10.1016/0014-5793(96)01046-0 8898100

[B25] Kato H Taniguchi Y Kurooka H Minoguchi S Sakai T Nomura-Okazaki S Tamura K Honjo T Involvement of RBP-J in biological functions of mouse Notch1 and its derivatives Development 1997 124 4133 4141 9374409 10.1242/dev.124.20.4133

[B26] Taylor MK Yeager K Morrison SJ Physiological Notch signaling promotes gliogenesis in the developing peripheral and central nervous systems Development 2007 134 2435 2447 10.1242/dev.005520 17537790 PMC2653864

[B27] Sonnenberg-Riethmacher E Miehe M Stolt CC Goerich DE Wegner M Riethmacher D Development and degeneration of dorsal root ganglia in the absence of the HMG-domain transcription factor Sox10 Mech Dev 2001 109 253 265 10.1016/S0925-4773(01)00547-0 11731238

[B28] Montelius A Marmigere F Baudet C Aquino JB Enerback S Ernfors P Emergence of the sensory nervous system as defined by Foxs1 expression Differentiation 2007 75 404 417 10.1111/j.1432-0436.2006.00154.x 17309606

[B29] Anderson DJ Lineages and transcription factors in the specification of vertebrate primary sensory neurons Curr Opin Neurobiol 1999 9 517 524 10.1016/S0959-4388(99)00015-X 10508743

[B30] Kurtz A Zimmer A Schnutgen F Bruning G Spener F Muller T The expression pattern of a novel gene encoding brain-fatty acid binding protein correlates with neuronal and glial cell development Development 1994 120 2637 2649 7956838 10.1242/dev.120.9.2637

[B31] Kageyama R Nakanishi S Helix-loop-helix factors in growth and differentiation of the vertebrate nervous system Curr Opin Genet Dev 1997 7 659 665 10.1016/S0959-437X(97)80014-7 9388783

[B32] Cho JH Tsai MJ The role of BETA2/NeuroD1 in the development of the nervous system Mol Neurobiol 2004 30 35 47 10.1385/MN:30:1:035 15247487

[B33] Morrow EM Furukawa T Lee JE Cepko CL NeuroD regulates multiple functions in the developing neural retina in rodent Development 1999 126 23 36 9834183 10.1242/dev.126.1.23

[B34] Gaiano N Nye JS Fishell G Radial glial identity is promoted by Notch1 signaling in the murine forebrain Neuron 2000 26 395 404 10.1016/S0896-6273(00)81172-1 10839358

[B35] Patten BA Peyrin JM Weinmaster G Corfas G Sequential signaling through Notch1 and erbB receptors mediates radial glia differentiation J Neurosci 2003 23 6132 6140 12853432 10.1523/JNEUROSCI.23-14-06132.2003 PMC6740346

[B36] Anthony TE Mason HA Gridley T Fishell G Heintz N Brain lipid-binding protein is a direct target of Notch signaling in radial glial cells Genes Dev 2005 19 1028 1033 10.1101/gad.1302105 15879553 PMC1091737

[B37] Britsch S Goerich DE Riethmacher D Peirano RI Rossner M Nave KA Birchmeier C Wegner M The transcription factor Sox10 is a key regulator of peripheral glial development Genes Dev 2001 15 66 78 10.1101/gad.186601 11156606 PMC312607

[B38] Okamura Y Saga Y Notch signaling is required for the maintenance of enteric neural crest progenitors Development 2008 135 3555 3565 10.1242/dev.022319 18832397

[B39] Danielian PS Muccino D Rowitch DH Michael SK McMahon AP Modification of gene activity in mouse embryos in utero by a tamoxifen-inducible form of Cre recombinase Curr Biol 1998 8 1323 1326 10.1016/S0960-9822(07)00562-3 9843687

[B40] Han H Tanigaki K Yamamoto N Kuroda K Yoshimoto M Nakahata T Ikuta K Honjo T Inducible gene knockout of transcription factor recombination signal binding protein-J reveals its essential role in T versus B lineage decision Int Immunol 2002 14 637 645 10.1093/intimm/dxf030 12039915

[B41] Soriano P Generalized lacZ expression with the ROSA26 Cre reporter strain Nat Genet 1999 21 70 71 10.1038/5007 9916792

[B42] Dai JX Hu ZL Shi M Guo C Ding YQ Postnatal ontogeny of the transcription factor Lmx1b in the mouse central nervous system J Comp Neurol 2008 509 341 355 10.1002/cne.21759 18512225

[B43] Brown NL Kanekar S Vetter ML Tucker PK Gemza DL Glaser T Math5 encodes a murine basic helix-loop-helix transcription factor expressed during early stages of retinal neurogenesis Development 1998 125 4821 4833 9806930 10.1242/dev.125.23.4821

[B44] Shindler KS Roth KA Double immunofluorescent staining using two unconjugated primary antisera raised in the same species J Histochem Cytochem 1996 44 1331 1335 10.1177/44.11.8918908 8918908

[B45] Kaufman MH The Atlas of Mouse Development 1992 London: Academic Press

[B46] OriginPro http://www-4.physik.uni-augsburg.de/exp5/computing/originpro.html

