# Supplementary figures and images for: The role of the transcription factor Rbpj in the development of dorsal root ganglia
Source: Neural Dev. 2011 Apr 21;6:14. doi: 10.1186/1749-8104-6-14 (PMC3110555; doi:10.1186/1749-8104-6-14)

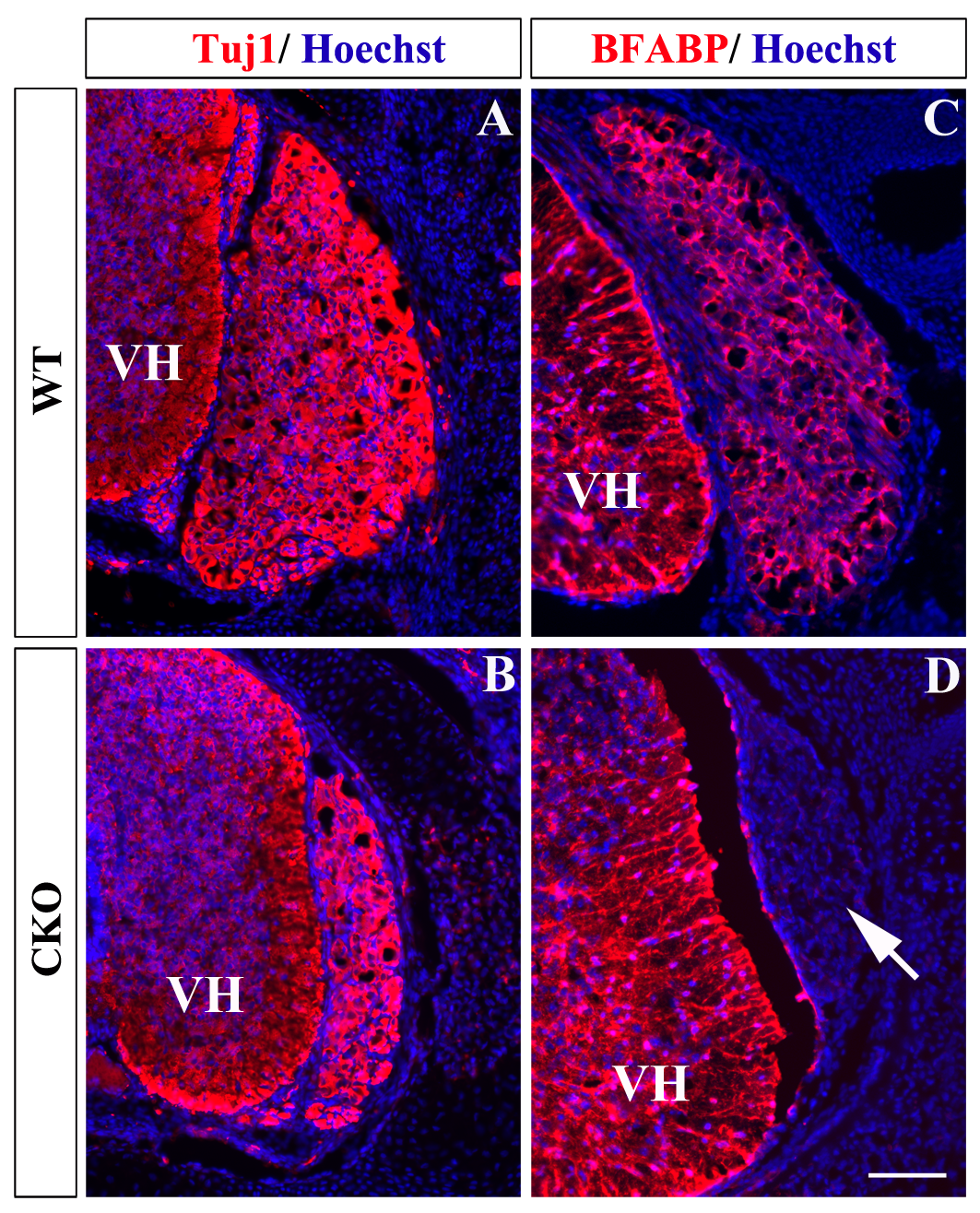

Supplement: Additional file 1 — Reduced number of neurons and near-complete loss of glia in Rbpj-deficient DRG at E16.5. (A-D) Tuj1 (A,B) and BFABP (C,D) immunostaining of transverse sections through wild-type and Rbpj-deficient DRG at E16.5 with Hoechst counterstaining. Note that BFABP expression is present in the spinal cord, but not in the DRG (arrow) of Rbpj CKO mice. VH, spinal ventral horn. Scale bars: 100 μm. [file 1749-8104-6-14-S1.TIFF]
